# Effects of Vibrotactile Feedback on Sedentary Behaviors in Adults: A Pilot Randomized Controlled Trial

**DOI:** 10.3390/ijerph16234612

**Published:** 2019-11-20

**Authors:** Makoto Nishimura, Hiroyuki Sasai, Yoshio Nakata, Seiji Maeda

**Affiliations:** 1 Graduate School of Comprehensive Human Sciences, University of Tsukuba, Tsukuba 305-8574, Japan; makoto.n.tyaym@gmail.com; 2 Graduate School of Arts and Sciences, The University of Tokyo, Tokyo 153-8902, Japan; 3 Research Team for Promoting Independence and Mental Health, Tokyo Metropolitan Institute of Gerontology, Tokyo 173-0015, Japan; 4 Faculty of Health and Sport Sciences, University of Tsukuba, Tsukuba 305-8574, Japan; nakata.yoshio.gn@u.tsukuba.ac.jp (Y.N.); maeda.seiji.gn@u.tsukuba.ac.jp (S.M.)

**Keywords:** sedentary behavior, vibrotactile feedback, objective monitoring

## Abstract

No effective and easily implemented intervention strategies for reducing sedentary behavior have been established. This pilot trial (UMIN000024372) investigated whether vibrotactile feedback reduces sedentary behavior. Twenty-six adults aged 30–69 years who were sedentary ≥8 h/day were randomly assigned to control (*n* = 13) or vibration (*n* = 13) groups. Participants wore a monitor 9 h daily for seven-day periods at baseline (week zero), during the intervention (weeks one, three, five, and seven), and after the intervention (week eight). During the eight-week intervention, vibration-group participants were notified by a vibration through the monitor whenever continuous sedentary time reached ≥30 min; they also received weekly reports of their sedentary patterns. Control-group participants did not receive feedback. The primary outcome was change in total sedentary time. Changes in longer bouts of sedentary time (≥35 min) were also assessed. No significant difference was found in the change in total sedentary time (control: −17.5 min/9 h, vibration: −9.1 min/9 h; *p* = 0.42). Although no significant differences were observed in sedentary time in longer bouts, vibration-group participants exhibited significantly lower sedentary time (–21.6 min/9 h, *p* = 0.045). Thus, vibration feedback does not appear to offer any advantages in reducing total sedentary time.

## 1. Introduction

Prolonged sitting has been associated with cardiovascular and all-cause mortality, as well as with various other adverse health outcomes (e.g., diabetes, cardiovascular diseases, and cancer) [1,2,3]. The Sedentary Behavior Research Network defined sedentary behavior as “any waking behavior characterized by an energy expenditure ≤1.5 metabolic equivalents (METs) while in a sitting or reclining posture” [4]. According to an international comparative study reporting the number of hours spent during a weekday in a sitting posture among adults in 20 countries, adults in Japan spent the longest time being sedentary (420 min/day) [5]. Thus, reducing sedentary behaviors is an important public health priority for the Japanese population.

Reducing sedentary behavior may be feasible through interventions that target sedentary behavior and physical activity [6]. According to meta-analytic evidence, activity-permissive workstations in which people can work while standing can reduce sedentary behavior in workdays by 77 min per 8 h in adults [7]. Use of the activPAL device (PAL Technologies, Glasgow, UK) is a validated method for assessing sedentary behavior under normal daily conditions [8]. One validation study reported that the activPAL correctly discriminated between sitting/lying and standing 95.9% of the time [9]. A function can be added to the activPAL that provides immediate vibration feedback when a wearer’s sedentary time continues for more than a certain period (e.g., 10 min, 30 min, or 60 min). According to a previous report of a sedentary behavior intervention study using this vibration feedback with full-time students, vibration feedback did not significantly change students’ total daily sedentary time, but it did decrease prolonged bouts (>30 min) of sedentary time [10].

To date, no effective intervention strategies that are easy for individuals to implement have been established for reducing sedentary behavior. Changing environmental factors, such as adding activity-permissive workstations or standing desks, is costly. Moreover, the effectiveness of using vibration feedback, such as that provided by the activPAL, in adults is unclear. In this study, the rationales for using the activPAL device were as follows: (1) its measurement accuracy of sedentary behavior has been well-recognized by the scientific community [9], (2) the outputs from this device can be easily compared with prior studies due to its widespread use [8,9,10], and (3) it has an added function of vibration feedback [10]. Therefore, the purpose of this pilot trial was to determine whether immediate vibrotactile feedback provided by an activity monitor worn on the thigh could reduce sedentary behavior in adults.

## 2. Methods

### 2.1. Study Design

This study was an eight-week randomized, controlled, pilot trial conducted at the Research Center of the University of Tsukuba between October and December 2016. Data were analyzed from January through November 2017. The study protocol involved three laboratory visits (an introductory orientation session, a baseline measurement, and a post-intervention measurement). The protocol was reviewed and approved by the ethical committee of the University of Tsukuba, Faculty of Health and Sport Sciences (approval number 28-62). This trial was registered in the University Hospital Medical Information Network Clinical Trials Registry (UMIN000024372) as of 12 October 2016. This article complies with the Consolidated Standards of Reporting Trials 2010 guideline [11] and its extension to randomized pilot and feasibility trials [12].

### 2.2. Participants and Randomization

Participants were recruited through advertisements in local newspapers. Eligibility criteria were an age of 30–69 years and a self-reported daily sedentary time of ≥8 h [13]. Exclusion criteria were (1) being currently pregnant or trying to conceive during the study period; (2) having irregular working hours, such as night-shift work; (3) having a scheduled disruption to the candidate’s lifestyle during the study period, such as an extended business trip; (4) currently participating or intending to participate in other clinical trials; and (5) being judged as otherwise ineligible by the principal investigator (S.M.). All participants provided written informed consent to participate before inclusion. Participants received financial compensation (10,000 JPY, equivalent to 92 USD in 2019) for completing this trial.

After stratifying intervention waves (two waves), participants were randomly assigned to either the control or the vibration group by computer-generated random numbers. In this trial, the second intervention wave was initiated one week after the first wave. A random number sequence was generated by an investigator (Y.N.) who had no contact with the participants, and the number sequence was maintained at a central, secure location until week one of each intervention wave.

### 2.3. Interventions

**Figure 1:**
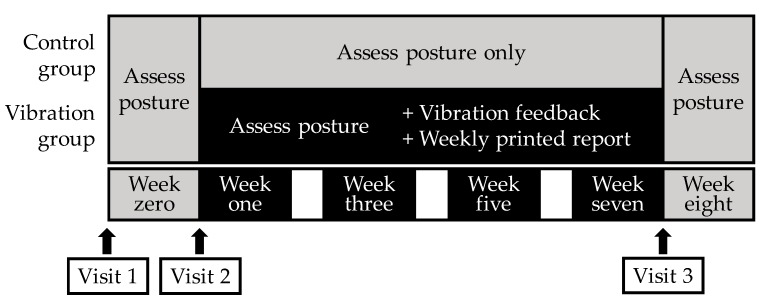
Schematic representation of the eight-week intervention.

Figure 1 shows the timeline for the procedures performed during the eight-week intervention. All participants wore the activPAL3VT monitor (PAL Technologies, Glasgow, UK) on the thigh for 9 h daily, from 9:00 a.m. to 6:00 p.m., for seven consecutive days at baseline (week zero), during the intervention (weeks one, three, five, and seven), and after the intervention (week eight). This trial adopted a 9-h time frame for wearing the monitor, which is shorter than that used in previous studies (typically 24 h) [14], because we were concerned about the risk of potential adverse effects, such as redness and itching, from the direct attachment of the monitor to the thigh for multiple weeks. Unlike standard seven-day observations of activPALs used in several population studies [15,16], adverse events associated with direct activPAL attachment were expected to occur more frequently in the present study due to multiple weeks of measurement. Considering that this trial is one of the first studies to use the activPAL monitor as a motivational tool, we prioritized feasibility, compliance, and safety concerns over internal validity, and thus decided to adopt a 9-h measurement period. The activPAL3VT device was attached to the front of the thigh using transparent film (Tegaderm^TM^, 3M Health Care, St. Paul, MN, USA), and was set to produce a small vibration when a wearer’s sedentary time reached a predefined duration (i.e., 30 min). During the intervention period, participants in the vibration group received immediate vibration feedback from the monitor when their continuous sedentary time reached 30 min [10,17]. They also received a printed weekly report of their recent sedentary patterns. Participants in the control group received neither vibrotactile feedback nor a weekly printed report; instead, their posture was assessed with the device (described in Section 2.4.1. below). At weeks zero and eight, no participant received vibration feedback; instead, their posture was assessed. The device and necessary instructional materials were mailed by post to minimize participant burden during the intervention period.

### 2.4. Measurements

#### 2.4.1. Sedentary Behavior and Posture Assessments

The primary outcome measure was a change in total sedentary time (min/9 h) from baseline values to the end of the intervention period (with total sedentary time taken as an average across weeks one to seven). Sedentary time was defined as time spent in a sitting/lying position, as recorded by the activPAL3VT device whenever it was parallel to the ground. The secondary outcome was a change in the number of transitions from sedentary to standing. The minimum definable sitting/upright time period in this study was the device default of 10 s, which is recommended by the manufacturer [14]. The device recorded standing time whenever it was stationary and perpendicular to the ground, and it recorded stepping time whenever it was moving and perpendicular to the ground. We defined changes in prolonged sedentary time (bouts lasting ≥30 min [10,17] and ≥35 min), standing time, and stepping time from baseline to the end of the intervention period as exploratory outcomes. In this study, during the intervention period, participants in the vibration group received immediate vibration feedback from the monitor when their continuous sedentary time reached 30 min. In previous studies, for sedentary bouts lasting ≥30 min, a person was not assessed as standing because it was calculated as sedentary bouts lasting ≥30 min even if he or she stood when the device vibrated. Therefore, we used a time period of 35 min to determine the effect of the intervention (i.e., to account for a person standing when the device vibrated). Data were obtained from two files saved on the devices: (1) a 15-s epoch summary file, from which we obtained the number of seconds spent in various activity states (i.e., sitting/lying, standing, and stepping) and the number of transitions from sitting/lying to standing, and (2) an “events” file, from which we obtained bouts of sedentary time. Participants self-reported in diaries the exact times they put on and took off the device. Records were defined as valid if they showed that the participant wore the device for at least 8 of 9 h per day, including on at least two weekdays and one weekend day. When records showed that the device was worn for <9 h, we calculated the number of minutes of sedentary behavior per hour, and then multiplied by nine to estimate min/9 h.

#### 2.4.2. Sociodemographic and Anthropometric Characteristics

We assessed sociodemographic characteristics, including age, sex, smoking status (smoker or non-smoker), educational attainment (college graduate or high school graduate or less), household income (<5 million JPY or ≥5 million JPY), marital status (married or unmarried), job description (full-time worker or not), and living arrangement (living alone or living with one or more other people), using a self-reported questionnaire and an additional face-to-face interview at the time of baseline measurement. Weight was measured, without shoes, to the nearest 0.1 kg using an InBody 770 (Biospace, Seoul, Korea). Height was measured to the nearest 0.1 cm on a wall-mounted stadiometer. Body mass index was determined using these measurements as weight (kg) divided by squared height (m^2^).

#### 2.4.3. Post-Intervention Survey

Participants were given a survey after they completed the study. The aim of this post-intervention survey was to obtain frank opinions from participants on the study intervention. The survey consisted of open-ended descriptive and selective questions and was filled out anonymously and voluntarily. Participants in both groups were asked to answer the following question using a four-point scale (“I was trying”, “I was trying a little”, “I was not really trying”, and “I was not trying at all”): “Were you trying to reduce sedentary behavior by wearing the device?”. Participants in the vibration group were asked to answer the following question using a four-point scale (“often vibrated”, “vibrated”, “did not vibrate too much”, and “hardly vibrated”): “Did the device vibrate?”, to answer the following question using a six-interval scale (100%, 80%, 60%, 40%, 20%, and 0%): “How often did you stand when the device vibrated?”, and to answer the following open-ended question: “Please describe your experience regarding the vibration frequency and standing on vibration.” Participants in the control group were asked to answer the following open-ended question: “Please describe any encumberment or inconvenience you experienced during the intervention period.”

### 2.5. Data Analysis

We set the target number of participants at 32. Since this was a pilot trial, there was no statistical basis for this sample size. We determined this target number because we believed that 32 participants would be adequate for obtaining a robust estimate of effect size, as described in another clinical trial [18]. Our primary analysis followed an intention-to-treat principle, and any missing data were replaced with the feasible and transparent “baseline observation carried forward” technique. Data were analyzed using IBM SPSS Statistics for Windows, Version 24.0 (IBM Corp., Armonk, NY, USA), with the level of statistical significance set at *p* = 0.05. Sociodemographic characteristics of the two groups were compared with independent-sample *t*-tests and chi-squared analyses. Primary (i.e., change in total sedentary time) and exploratory outcomes (i.e., change in prolonged sedentary time [bouts lasting ≥35 min]) were compared using a two-way (group: Control and vibration; time: Week zero and weeks one to seven) repeated-measures analysis of variance. A main time effect with ANOVA was used to interpret within-group differences independent of group allocation. Effect size calculations were also used to describe the magnitude of differences in total sedentary behavior time and prolonged bouts of sedentary time (≥35 min) within groups using Cohen’s d. Changes in secondary and exploratory outcomes are presented with means and 95% confidence intervals (CIs).

## 3. Results

**Figure 2:**
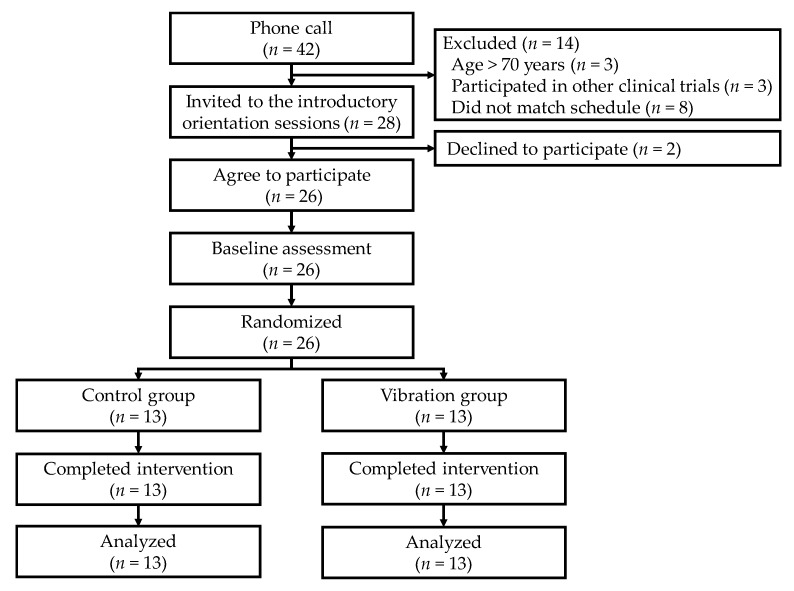
Participant flow from recruitment to end of trial.

Figure 2 shows a participant flowchart. We had 42 phone contacts, in which we briefly screened interested participants and eventually invited 28 candidates to the introductory orientation sessions. We obtained written informed consent from all 26 participants (19 women and seven men) who ultimately agreed to participate. They were randomly assigned to either the control or vibration group. All 26 participants (control: *n* = 13; vibration: *n* = 13) completed the eight-week study.

**Figure 3:**
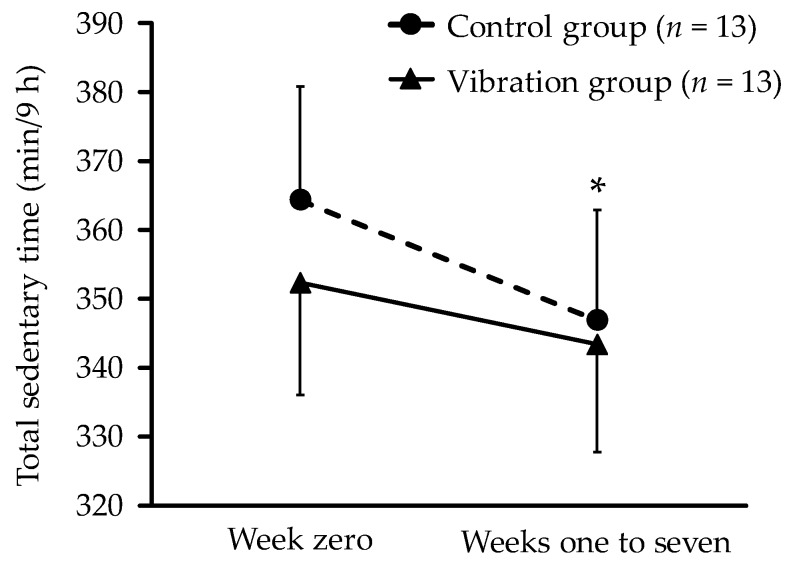
Change in sedentary time from week zero to weeks one to seven. Each data point represents mean value for all participants, with missing data replaced by baseline observation carried forward. Error bars indicate standard error. * *p* < 0.05 for within-group differences.

Table 1 shows the sociodemographic characteristics of the participants at baseline. There were no notable differences between the two groups. The pattern of change in sedentary time is shown in Figure 3. No significant interaction effect (F = 0.67, *p* = 0.42) or group effect on sedentary time was observed; however, a significant time effect (F = 6.74, *p* = 0.016) was observed. The mean sedentary time in the control group declined significantly, by 17.5 min/9 h (95% CI: −32.4 to −2.5 min; Cohen’s d = 0.64) from week zero to weeks one to seven. The mean (standard deviation) sedentary time at week eight in the control and vibration groups was 345 (76) min/9 h and 339 (64) min/9 h, respectively.

**Figure 4:**
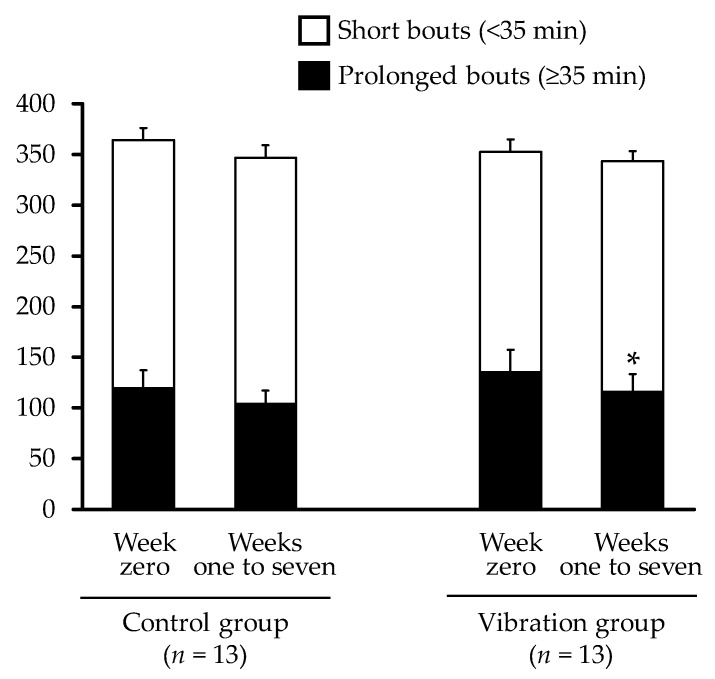
Changes in prolonged (≥35 min) and short (<35 min) bouts of sedentary time from week zero to weeks one to seven. Each data point represents mean value for all participants, with missing data replaced by baseline observation carried forward. Error bars indicate standard error. * *p* < 0.05 for within-group differences.

The pattern of changes in prolonged sedentary time is shown in Figure 4. No significant interaction effect (F = 0.350, *p* = 0.560) or group effect was observed for sedentary time; however, a significant time effect (F = 5.791, *p* = 0.024) was observed. The mean prolonged bout of sedentary time (≥35 min) in the vibration group declined significantly by 21.6 min/9 h (95% CI: −42.7 to −0.6; d = 0.59) from week zero to weeks one to seven. The mean (standard deviation) prolonged bout of sedentary time (≥35 min) at week eight in participants in the control and vibration groups was 103 (48) min/9 h and 111 (63) min/9 h, respectively.

Table 2 shows the changes in posture measurements. There were no significant between-group differences for any measurements. The change from week zero to weeks one to seven in standing time increased significantly within the control group

Twenty-one participants (81%; control: *n* = 10, vibration: *n* = 11) answered the post-intervention survey. Of those, 15 participants (71%) agreed with the question, “Were you trying to reduce sedentary behavior by wearing the device?”. Of 11 respondents in the vibration group, three participants responded, “did not vibrate too much” or “hardly vibrated”. Of the eight respondents who answered “often vibrated” or “vibrated” in the vibration group, three participants reported standing for less than half of the vibrations. Among the 10 respondents in the control group, half reported being burdened or irritated by adverse effects, such as redness and itching.

## 4. Discussion

The purpose of this pilot study was to determine whether immediate vibrotactile feedback from a thigh-worn activity monitor could reduce sedentary behavior in adults. We found no significant between-group differences in changes in total sedentary time. However, participants receiving vibrotactile feedback exhibited significantly fewer minutes of sedentary time accumulated in longer bouts (≥35 min).

In this study, there was no significant difference between groups during the vibration intervention period with respect to total sedentary time. Responses to the post-intervention survey from the vibration group included the observation that vibrations occurred in situations in which it was not possible to stand up (e.g., while driving or during a business meeting). The number of transitions from sitting/lying to standing at week zero in the vibration group was 40 times per 9 h, or ~4.5 times per hour. This corresponds to approximately 13 min between transitions, which is more frequent than what would have been prompted by the device when recommending participants to stand from sitting/lying. This might be another explanation for the lack of difference in total sedentary time between groups.

Two possible explanations for reduced sedentary time in the control group should be considered. A systematic review that examined the effect of the use of pedometers reported that using pedometers increased steps by about 2000 [19]. This suggests the possibility that the total sedentary time in the control group decreased significantly in this study simply due to the provision of activPAL devices. Additionally, it is possible that results were influenced by the Hawthorne effect, which is a change in the behavior of research participants based on their motivation to respond to the expectations of the researchers supervising the study. It may be that either or both of these effects influenced the control group.

In public health promotion, recent attention has been paid not only to total sedentary time but also to both the accumulated sedentary time in continuous extended bouts and the interruption of prolonged sedentary time. It has been reported that the longer a person sits continuously, the higher the risk of mortality [20], obesity, and postprandial hyperglycemia; these risks can all be reduced by interrupting prolonged bouts of sedentary behavior with low-intensity physical activity [21]. In this study, participants receiving vibrotactile feedback exhibited significantly fewer minutes of sedentary time accumulated in longer bouts (≥35 min). This result is in accordance with the findings of the previously described intervention that used the vibration function of the activPAL monitor in full-time college students and found that it significantly reduced prolonged bouts of sedentary time (≥30 min) in the intervention group [10].

Our study has several limitations. First, since this was a pilot trial, there was no statistically determined basis for the sample size. Thus, it is possible that the number of participants was insufficient to detect significant differences. Second, participants were asked to wear the device for seven-day periods, from 9 a.m. to 6 p.m. (after taking into consideration the incidence of adverse effects such as redness and itching), so posture was only assessed during the time specified. Third, participants were allowed to stop the vibration of the device temporarily when they knew that they would be sitting for a long time and could not stand up (e.g., because they were in a meeting or were driving for a long time). Thus, we could not determine if the device vibrated consistently for all participants.

Adverse effects have been reported, such as redness and itching, from the attachment of the device directly to the thigh [22]. The post-intervention survey demonstrated that half of the 10 respondents in the control group reported being burdened or irritated by such adverse effects. Of the 26 participants, 19 (73.1%) reported redness at least once during weeks zero and eight in their diaries. It has been reported that placing the device in a pouch and wrapping it around the thigh with a stretchy band also allows the device to operate effectively [23]. Therefore, as a method that can avoid adverse effects, avoiding direct attachment of the device to the skin, or using tape with better breathability may be beneficial for future research.

The strengths of this study include the randomized controlled trial research design based on CONSORT 2010 guidelines [11] and the use of the activPAL device, which is a validated method for evaluating sedentary behavior. There have been few intervention studies using the vibration function of the activPAL device [10], and this is the first such study targeting adults in Japan.

There are several noteworthy implications for future trials on a larger scale. First, it may be effective to set the timing of the added vibration function of activPAL according to individually assessed sedentary bout distribution before and during an intervention. And individualized cutoff setting for activating the vibration feature may enhance the effectiveness of an intervention that aims to reduce sedentary behavior. Second, we adopted a reduced wearing time from 9:00 a.m. to 6:00 p.m., and many participants were probably working during that time. Assessing sedentary behavior for 24 h may more clearly capture the beneficial effects of the vibration function on sedentary behavior during leisure time, such as television watching and relaxing on a couch. These are considered representative forms of sedentary behavior in modern societies. Third, we instructed participants to stand for at least 1 min whenever the device vibrated. If bouts of sedentary behavior are interrupted for 5 min or longer by standing or walking, greater health and work-productivity benefits can be expected. For that purpose, it is necessary to assess various newly proposed sedentary and activity metrics (e.g., sedentary-to-active transition probabilities) [24] and health and work productivity outcomes. Based on the lessons learned from this study, we plan to refine the intervention program and design another small randomized controlled trial to obtain a robust estimate of effect size; this will be utilized to conduct a larger trial in the future.

As an implication for real-world practice, if posture detection by wearable devices and/or smartphones placed in the pocket becomes widely disseminated, sedentary-reducing interventions with vibration functions could be promising. These may offer a feasible option for reducing sedentary behavior and promoting health for people who are not interested in participating in regular exercise or sports.

## 5. Conclusions

This study suggests that a vibrotactile feedback program does not offer any advantages over a non-feedback control for reducing sedentary behavior. In future research, setting individualized cutoffs for vibrotactile feedback (e.g., 10 min or 20 min) may more effectively reduce sedentary behavior.

## Figures and Tables

**Table 1:** Participant sociodemographic characteristics at baseline.

Characteristics	Control (*n* = 13)	Vibration (*n* = 13)	Total (*n* = 26)
Age, years	51 (9)	51 (10)	51 (9)
Women, n (%)	8 (62)	11 (85)	19 (73)
Current smoker, n (%)	1 (8)	0 (0)	1 (4)
College graduate, n (%)	7 (54)	6 (46)	13 (50)
Household income ≥5 million JPY *, n (%)	11 (85)	5 (38)	16 (62)
Married *, n (%)	9 (69)	6 (46)	15 (58)
Full-time worker, n (%)	10 (77)	10 (77)	20 (77)
Living with one or more others *, n (%)	11 (85)	11 (85)	22 (85)
Weight, kg	57.6 (9.3)	52.8 (8.4)	55.2 (9.0)
Body mass index, kg/m^2^	21.8 (3.0)	20.5 (2.1)	21.1 (2.6)

Data presented as mean (standard deviation) for continuous variables and number (%) for categorical variables. * Data only available for 25 participants (13 in control group and 12 in vibration group).

**Table 2:** Changes in exploratory behavioral outcomes during the eight-week intervention period.

Outcomes	Control (*n* = 13)	Vibration (*n* = 13)	*p* Values
Transitions from sedentary to standing, times/9 h			
Week zero	40 (11)	46 (19)	
Weeks one to seven	39 (6)	46 (17)	
Change	−0.4 (−4.3, 3.6)	0.6 (−3.4, 4.5)	0.73
≥30-minute bouts sedentary time, min/9 h			
Week zero	151 (70)	142 (84)	
Weeks one to seven	136 (52)	121 (69)	
Change	−14.9 (−36.7, 7.0)	−20.5 (−42.4, 1.3)	0.71
Standing time, min/9 h			
Week zero	117 (48)	137 (55)	
Weeks one to seven	133 (47)	144 (53)	
Change	15.9 (3.1, 28.7)	7.5 (−5.3, 20.2)	0.35
Stepping time, min/9 h			
Week zero	59 (18)	51 (11)	
Weeks one to seven	65 (17)	55 (10)	
Change	5.9 (−1.7, 13.5)	3.9 (−3.6, 11.5)	0.71

Data were presented as mean (standard deviation) or mean (95% confidence interval).
